# Supplementary zinc and vitamin D in management of symptomatic oral lichen planus: a three-arm randomized clinical trial

**DOI:** 10.1186/s12903-025-06173-1

**Published:** 2025-06-02

**Authors:** Alaa Aboushousha, Yasmine Kamal, Shereen Ali

**Affiliations:** Faculty of Dentistry, Cairo University, 11 El-Saraya Street, Manial, Cairo, 11553 Egypt

**Keywords:** Oral lichen planus, Zinc, Vitamin D, Corticosteroids, Triamcinolone acetonide, Clinical trial

## Abstract

**Background:**

Oral lichen planus (OLP) is a chronic inflammatory disease affecting the oral mucosa, with immune mediated pathogenesis. Both zinc and vitamin D are essential micronutrients implicated in OLP pathogenesis, with zinc influencing epithelial growth, wound healing, and oxidative stress mitigation, while vitamin D modulates immune cell function, cytokine activity, and keratinocyte behavior. The current trial was conducted to assess the effect of topical corticosteroid supplemented with zinc or vitamin D compared to topical corticosteroid in management of symptomatic oral lichen planus.

**Methods:**

Forty-two patients diagnosed with symptomatic oral lichen planus (atrophic, erosive, or bullous types) were included in the study, based on clinical examination and histopathological confirmation in accordance with the modified World Health Organization (WHO) criteria (2003). Participants were divided randomly into three parallel groups. Group I received topical corticosteroid; Group II received topical corticosteroid with systemic zinc; Group III received topical corticosteroid with systemic vitamin D. The outcome measures included pain intensity using Visual Analogue Scale and clinical improvement using Thongprasom et al. scoring system. Treatment lasted for 8 weeks; patients were clinically evaluated at baseline and on weekly basis.

**Results:**

Intergroup comparisons revealed that by Week 3, pain scores were significantly lower in Groups II and III compared to Group I. Intragroup analysis showed significant reductions in pain scores over time for all groups, with Group II showing the most pronounced decrease from baseline to Week 7. Thongprasom scores decreased significantly in all groups, with Group II showing the most significant reduction by Week 8.

**Conclusions:**

Systemic zinc supplementation combined with topical corticosteroid can be used as an adjunct therapy for OLP management. Topical corticosteroid supplemented with vitamin D has comparable effect to topical corticosteroid alone on pain and clinical improvement in symptomatic OLP.

**Clinical trial registration:**

NCT04765267 (full protocol can be accessed). Date of Initial Clinical Trial Registration: 4 February 2021.

## Background

Oral lichen planus (OLP) is a chronic inflammatory disease affecting the oral mucosal, mediated by T cells with undetermined pathogenesis [1]. According to WHO clinical criteria, oral lichen planus (OLP) presents as white and red multifocal lesions with symmetric, often bilateral distribution and may appear in reticular, papular, plaque-like, erosive, atrophic, or bullous forms. Lesions commonly affect the buccal mucosa, tongue, and gingiva, where gingival involvement may present as desquamative gingivitis, often resembling other oral mucosal conditions. The atrophic and erosive types are typically symptomatic with greater patient discomfort and higher risk of malignant transformation which requires active clinical management. [2]. The World Health Organization (WHO) classified oral lichen planus (OLP) as a potentially malignant disorder in 2005, with reported malignant transformation rates ranging from 0.4% to 12.5%, averaging around 1.09% [3]. Genetic alterations associated with malignant progression in OLP include chromosome 9 monosomy, P53 and PCNA overexpression, c-erbB2 loss, and elevated telomerase activity [4, 5]. The risk of transformation is influenced by lesion severity, duration, patient demographics, and geographic factors [3–5].

Accurate assessment of disease severity is crucial for diagnosis, monitoring progression, and evaluating treatment efficacy [6]. Several scoring systems have been developed to objectively quantify lesion severity and patient-reported symptoms [7, 8]. Chaitanya et al. recently introduced a novel, multidimensional scoring system aimed at enhancing the clinical assessment of oral lichen planus by addressing its multifactorial nature. The proposed framework evaluates the condition across four primary axes: clinical presentation, psychological stress, histopathological findings, and anatomical site involvement. The clinical axis classifies lesions from asymptomatic reticular types to those with erosive or potentially malignant features. The psychological axis incorporates stress as a contributory factor in disease progression. Histopathological axis spans a spectrum from mild basal cell degeneration to carcinoma in situ. Finally, the site involvement axis gives greater significance to lesions affecting high-risk areas such as the tongue and floor of the mouth, as well as those with extraoral manifestations. Collectively, this comprehensive model offers a structured approach to track disease activity, predict outcomes, and guide individualized treatment planning [9].

The management of oral lichen planus (OLP) remains challenging, with treatment focused on symptoms control and inflammation reduction rather than cure [10]. Topical, injectional, or systemic corticosteroids remain the gold standard, though their use is limited by side effects which are associated with the use of corticosteroids in all of their available forms, with oral candidiasis being the most frequently reported [11, 12]. In refractory cases, immunomodulators and alternative therapies—including vitamins, trace elements, and herbal or laser-based treatments—have shown promise [13–15].

Micronutrients are the cornerstones in the maintenance of biodynamic of the body. The progression of various diseases can be attributed to either deficiency or excess of micronutrients. Micronutrient deficiency dysregulates the balanced host natural defense response by suppressing immune functions through affecting the innate (physical barriers in skin/mucosa), cellular, and humoral immunity systems. They generally play an important role in the regenerative processes against oxidative stress products in the tissues [13]. Many investigators observed reduced levels of micronutrients as iron, zinc, calcium, vitamin B12, folic acid, vitamin C, vitamin D, and vitamin E in several inflammatory disorders, including OLP [16, 17].

Zinc and vitamin D have both emerged as promising adjunct therapies in the management of oral lichen planus (OLP) due to their combined anti-inflammatory, antioxidant, and immunomodulatory properties. Zinc plays a critical role in epithelial repair and immune regulation, acting as a cofactor in wound healing enzymes and antioxidant systems such as superoxide dismutase. It supports T-cell–mediated immunity and mitigates oxidative stress, a key factor implicated in OLP pathogenesis [18–21]. Similarly, vitamin D regulates immune responses by suppressing Th1 and Th17-mediated inflammation and enhancing T-regulatory cell activity. It also inhibits macrophage overactivation and reduces the expression of inflammatory mediators such as IL-2, TNF-α, and MMP-9 in keratinocytes, thereby promoting epithelial stability [22, 23]. Moreover, psychological stress—a known trigger of OLP—can disrupt vitamin D metabolism by downregulating its receptors, further supporting the relevance of supplementation in affected individuals [24]. These complementary mechanisms position both micronutrients as biologically plausible agents for enhancing the therapeutic outcomes of conventional corticosteroid treatment in OLP.

The aim of the current trial is to assess the effect of topical corticosteroid augmented with zinc or vitamin D compared to topical corticosteroid on pain and clinical improvement in symptomatic OLP. Based on the potential immunomodulatory and anti-inflammatory effects of zinc and vitamin D, it is hypothesized that their adjunctive use alongside topical corticosteroids will enhance therapeutic outcomes in patients with symptomatic oral lichen planus.

## Methods

### Trial design

This is a randomized clinical trial (RCT) parallel group, three arm, superiority framework with 1:1:1 allocation ratio. This clinical trial was conducted after the approval of the ethical committee of Faculty of Dentistry, Cairo University, Egypt, in September 2020 (Code: 23920). Informed consent to participate was obtained from all participants in the study. The protocol registered at ClinicalTrials.gov by number NCT04765267. The trial followed the principles of the Helsinki Declaration [25]. This study was conducted in accordance with the CONSORT guidelines to ensure transparency and completeness in reporting.

### Participants

The study was conducted in the Oral Medicine Clinics at the Faculty of Dentistry, Cairo University, Egypt. Inclusion criteria included patients diagnosed with symptomatic OLP (atrophic, erosive, or bullous types) regardless of pain severity. Diagnosis was based on clinical examination and histopathological confirmation according to the modified WHO criteria (2003) for the diagnosis of OLP [26]. Patients had to be free from any visible oral lesions other than OLP, agreed to take the supplied interventions, agreed to participate in the study, and accepted to sign the informed consent. Exclusion criteria included patients suffering from any systemic disease, patients on any systemic treatment at least eight weeks prior to the trial, patients on any oral topical medications for at least four weeks prior to the study, and pregnant and lactating women.

### Interventions

Group I received topical Corticosteroid (Kenacort A Orabase: triamcinolone acetonide (TA) 0.1% adhesive paste – Dermapharm) four times daily for 8 weeks. Group II received the same corticosteroid agent (Kenacort A Orabase) four times daily, with systemic zinc (Octozinc: Zinc sulphate heptahydrate 25 mg tablets – October Pharma) twice daily, for 8 weeks. Group III received the same corticosteroid agent (Kenacort A Orabase) four times daily, with systemic vitamin D (Vidrop 15 ml oral solution: Cholecalciferol “vitamin D3” 2800 IU/ml - Medical Union Pharmaceuticals) four drops daily, for 8 weeks. According to the manufacturer, each 1 ml of ‘Vidrop oral solution’ is equal to 28 drops of Vitamin D3 (Cholecalciferol), while each drop contains 100 IU of vitamin D3. So, patients were asked to consume 4 drops daily (400 IU/day which is equivalent to 2800 IU/week), for 8 weeks. After completing four-week of topical corticosteroids applications, all patients in the trial received a prescription for topical oral antifungal treatment (Daktarin 2% oral gel – miconazole), applied 4 times daily for one week, alongside the previously assigned interventions in each group. This was done to prevent any secondary infections with oral candidiasis. Throughout the trial, patients were required to refrain from using any other topical or systemic medications. They were scheduled for weekly follow-up appointments over the course of eight weeks.

## Outcomes

### Primary outcome

The primary outcome was to assess pain intensity, using Visual Analogue Scale VAS (0–10), a widely accepted and validated unidimensional tool frequently used in clinical research involving oral mucosal lesions such as oral lichen planus (OLP). The VAS consists of a 10-centimeter horizontal line ranging from 0, indicating no pain, to 10, representing the worst imaginable pain [27]. Patients were instructed to mark a point on the scale that best represented their perceived level of discomfort, allowing for a subjective yet quantifiable measurement of pain. This method has demonstrated reliability and sensitivity in detecting pain variation among OLP patients [28]. Pain assessments were conducted at baseline, daily during the first week, and then weekly over the following seven weeks, enabling a detailed evaluation of treatment response over time.

### Secondary outcome

The secondary outcome was clinical improvement which was measured using Thongprasom et al. scoring system at baseline, and weekly for the eight weeks [29]. *Thongprasom scoring system*, remains one of the most widely used and validated tools for grading OLP severity [29]. This scoring system classifies lesions on a scale from 0 to 5 based on the presence and extent of white striae, erythematous (atrophic) areas, and ulcerative (erosive) lesions. A score of 0 represents normal mucosa; 1 indicates the presence of white striae only; 2 corresponds to white striae with an atrophic area less than 1 cm^2^; 3 denotes white striae with atrophic area greater than 1 cm^2^; 4 indicates white striae with erosive area less than 1 cm^2^; and 5 signifies white striae with erosive area greater than 1 cm^2^. The simplicity, reproducibility, and applicability of the Thongprasom score make it a valuable tool for both clinical and research settings [29]. Lesion size was measured using a calibrated periodontal probe, which allowed for accurate estimation of lesion dimensions during clinical examination. Photographical records of patients were taken at baseline, and weekly for the eight weeks.

### Sample size

Sample size calculation was based on the previous study by Suvarna et al. [30], It was determined using the following data: primary outcome as burning sensation using VAS, value for outcome (mean & SD): 1.45 ± 0.826. Statistical test used for calculation was Independent samples t-test, alpha level of significance 5%, and power 80 %. The calculated sample size was 12 subjects per group, and 20 % increase for anticipated missing data, making the final sample size 14 subjects per group, 42 subjects as total.

### Randomization and blinding

Simple randomization was generated using a computerized random number generator (random.org) with an allocation ratio of 1:1:1. Allocation concealment was achieved by using sequentially numbered opaque sealed envelopes until the interventions were assigned. The statistician generated the randomization, while SA carried out the allocation concealment. Participant enrollment was conducted by AA, and YK assigned the interventions. The current trial is a double-blinded trial where both the outcome assessor AA and statistician were blinded. However, it was not possible to blind the participants due to the differing nature and formulation of the interventions.

### Statistical methods

Numerical data were explored for normality by checking the distribution of data and using tests of normality (Kolmogorov-Smirnov and Shapiro-Wilk tests). Age data showed normal (parametric) distribution while pain (VAS) and Thongprasom scores showed non-normal (non-parametric) distribution. Data were presented as median, range, mean and standard deviation (SD) values. For parametric data; one-way ANOVA test was used to compare between mean age values in the three group. For non-parametric data; Kruskal-Wallis test was used to compare between the three groups. Friedman’s test was used to study the changes within each group. Dunn’s test was used for pair-wise comparisons when Kruskal-Wallis or Friedman’s test is significant. Qualitative data were presented as frequencies and percentages. Fisher’s Exact test was used to compare between the three groups. The significance level was set at P ≤ 0.05. Statistical analysis was performed with IBM SPSS Statistics for Windows, Version 23.0. Armonk, NY: IBM Corp.

## Results

**Fig. 1:**
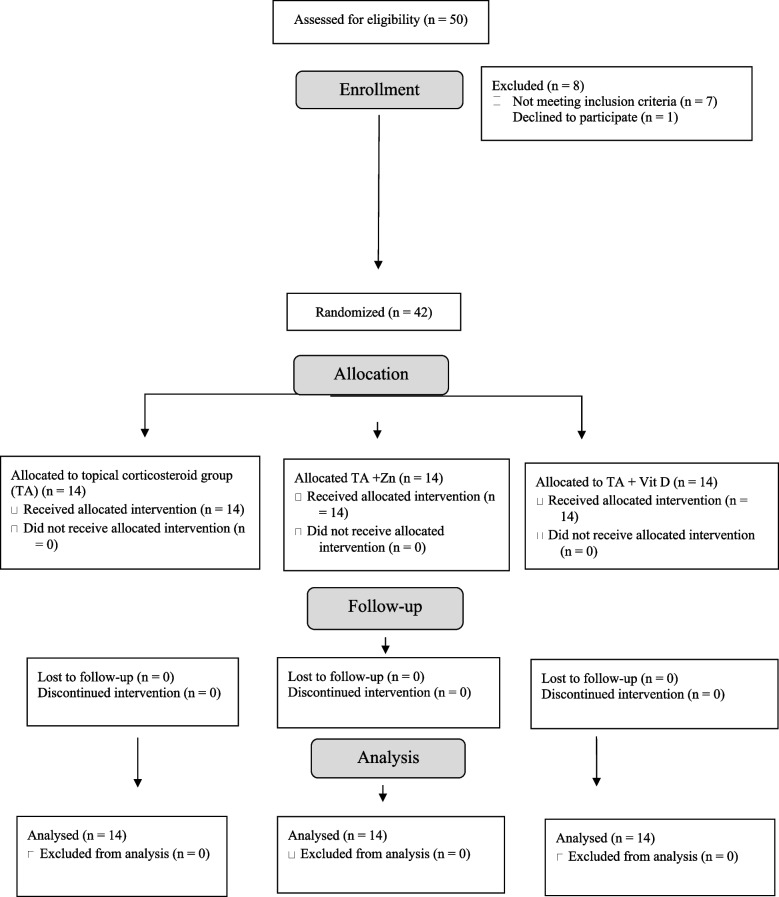
CONSORT flow diagram

A total of 50 patients were screened, of whom 42 met the eligibility criteria and were enrolled consecutively between April 2021 and September 2023. Eight were excluded due to systemic diseases (*n* = 5), ongoing topical therapy (*n* = 2), or refusal to participate (*n* = 1). No participants dropped out, and no adverse effects were observed in either group. All data were analyzed (Fig. 1).

### Base line characteristics

The three groups were comparable in age and gender (*p* = 0.900 and *p* = 1.000, respectively) (Table 1).

**Table 1:** Mean, standard deviation (SD), frequencies (n), percentages, results of one-way ANOVA test and Fisher’s Exact test for comparison between base line characteristics in the three groups

	Group I (TA) (*n* = 14)	Group II (TA + Zn) (*n* = 14)	Group III (TA + VIT D) (*n* = 14)	*P*-value
Age (Years)				0.900
Mean (SD)	50.8 (11.4)	52.6 (13.4)	53.1 (17.4)	
Gender [n (%)]				1
Male	4 (28.6)	5 (35.7)	5 (35.7)	
Female	10 (71.4)	9 (64.3)	9 (64.3)	

*TA* topical Triamcinolone acetonide, *Zn* systemic zinc, *VIT D* systemic vitamin D

^*^Significant at* P* ≤ 0.05

### Pain (VAS) score

There were no significant differences in baseline pain scores across groups. However, by Week 3, pain scores were significantly lower in Groups II (TA + Zn) and III (TA + Vit D) compared to Group I (TA only), with median scores of 3 in both groups versus 5 in Group I (*p* = 0.015). Differences persisted in favor of Groups II and III through Week 8, though not always reaching statistical significance (Table 2).

**Table 2:** Comparison of pain (VAS) scores between the three groups and within each group

Time	Group I (TA) (*n* = 14)		Group II (TA + Zn) (*n* = 14)		Group III (TA + VIT D) (*n* = 14)		*P*-value	*Effect size (Eta squared)*
	Median (Range)	Mean (SD)	Median (Range)	Mean (SD)	Median (Range)	Mean (SD)		
Baseline	9 (5–10) ^A^	8.79 (1.58)	9 (5–10) ^A^	8.57 (1.79)	8 (3–10) ^A^	7.86 (2.35)	0.530	0.044
Day 1	8 (3–10) ^AB^	8 (1.84)	8.5 (4–10) ^AB^	8 (1.84)	8 (1–10) ^AB^	6.93 (2.56)	0.417	0.058
Day 2	8 (3–10) ^B^	7.64 (1.91)	7 (4–10) ^BC^	7.29 (1.77)	7 (1–10) ^B^	6.57 (2.47)	0.421	0.047
Day 3	7 (3–10) ^B^	7.14 (1.99)	7 (3–9) ^C^	6.64 (1.69)	6 (1–9) ^C^	5.64 (2.41)	0.253	0.091
Day 4	6.5 (3–10) ^C^	6.71 (2.05)	7 (3–9) ^C^	6.71 (1.73)	5 (1–9) ^C^	5.43 (2.41)	0.256	0.084
Day 5	6 (2–10) ^C^	6.14 (2.35)	6 (3–8) ^D^	5.69 (1.7)	5 (1–8) ^CD^	4.79 (2.19)	0.322	0.073
Day 6	5 (2–9) ^D^	5.64 (2.53)	5.5 (3–7) ^D^	5.36 (1.55)	4.5 (1–7) ^D^	4.29 (2.09)	0.303	0.077
Day 7/Week 1	5 (2–9) ^D^	5.5 (2.71)	4.5 (3–7) ^E^	4.93 (1.59)	5 (1–7) ^D^	4 (2.18)	0.331	0.078
Week 2	5 (2–9) ^D^	5.79 (2.26)	4 (2–7) ^E^	4.29 (1.59)	4.5 (0–6) ^D^	3.71 (1.98)	0.073	0.176
Week 3	5 (0–8) ^Da^	5 (2.25)	3 (0–6) ^Fb^	2.93 (1.82)	3 (0–5) ^Eb^	2.69 (2.06)	0.015*	0.218
Week 4	4 (0–9) ^DE^	4.71 (2.84)	2 (0–8) ^F^	2.71 (2.4)	2 (0–5) ^E^	2.21 (2.08)	0.055	0.172
Week 5	5 (0–9) ^E^	3.85 (3.08)	1.5 (0–5) ^FG^	1.79 (1.85)	2 (0–5) ^EF^	2.07 (2.02)	0.157	0.136
Week 6	3.5 (0–9) ^E^	3.64 (2.9)	1 (0–5) ^G^	1.79 (1.85)	1 (0–5) ^F^	1.71 (2.02)	0.138	0.140
Week 7	1.5 (0–9) ^F^	2.93 (3.25)	0 (0–5) ^G^	1 (1.62)	0.5 (0–5) ^F^	1.57 (2.06)	0.238	0.108
Week 8	3.5 (0–9) ^E^	3.36 (3.23)	0 (0–5) ^G^	1.21 (2.12)	0 (0–5) ^F^	1.51 (2.1)	0.067	0.131
*P*-value	< 0.001*		< 0.001*		< 0.001*			
Effect size (w)	0.607		0.888		0.861			

Different (uppercase letters) superscripts in the same column indicate statistically significant change by time, Different (lowercase letters) superscripts in the same row indicate statistically significant difference between groups

*TA* topical Triamcinolone acetonide, *Zn* systemic zinc, *VIT D* systemic vitamin D

^*^Significant at *P* ≤ 0.05

Within-group analysis showed a significant reduction in VAS scores over time in all groups (*p* < 0.001), with Groups II and III achieving greater and more sustained pain reduction.

### Thongprasom score

Baseline clinical scores were similar across groups. By Week 8, the median scores declined to 1.5 (Group I), 0 (Group II), and 1 (Group III), with a trend toward significance (*p* = 0.073). All groups showed significant intra-group reductions from baseline to Week 8 (*p* < 0.001), with the greatest improvement observed in Group II (Table 3).

**Table 3:** Comparison between clinical (Thongprasom) scores between the three groups and within each group

Time	Group I (TA) (*n* = 14)		Group II (TA + Zn) (*n* = 14)		Group III (TA + VIT D) (*n* = 14)		*P*-value	*Effect size (Eta squared)*
	Median (Range)	Mean (SD)	Median (Range)	Mean (SD)	Median (Range)	Mean (SD)		
Base line	5 (2–5) ^A^	4.36 (1.08)	5 (2–5) ^A^	4.07 (1.21)	3.5 (2–5) ^A^	3.86 (1.1)	0.447	0.034
Week 1	5 (2–5) ^A^	4.14 (1.1)	3.5 (2–5) ^B^	3.71 (1.27)	3 (1–5) ^A^	3.29 (1.14)	0.168	0.088
Week 2	4 (1–5) ^B^	3.57 (1.16)	3.5 (1–5) ^B^	3.43 (1.45)	3 (1–5) ^A^	3.21 (1.12)	0.702	0.015
Week 3	3.5 (1–5) ^B^	3.21 (1.12)	2 (1–5) ^C^	2.36 (1.45)	3 (1–5) ^AB^	2.71 (1.27)	0.182	0.075
Week 4	2.5 (1–5) ^C^	2.93 (1.38)	2 (1–4) ^C^	2.14 (1.17)	2 (1–5) ^B^	2.36 (1.39)	0.264	0.064
Week 5	2.5 (1–5) ^C^	2.64 (1.45)	1.5 (1–4) ^C^	1.93 (1.21)	2 (1–4) ^B^	2 (0.96)	0.310	0.069
Week 6	2 (1–5) ^C^	2.5 (1.45)	1 (1–4) ^C^	1.86 (1.23)	1.5 (1–4) ^B^	1.79 (0.97)	0.262	0.068
Week 7	2 (1–5) ^C^	2.21 (1.48)	1 (1–3) ^C^	1.43 (0.76)	1 (1–4) ^B^	1.71 (0.99)	0.266	0.083
Week 8	1.5 (0–5) ^C^	1.86 (1.61)	0 (0–3) ^D^	0.71 (1.14)	1 (0–3) ^B^	1.29 (1.14)	0.073	0.119
*P*-value	< 0.001*		< 0.001*		< 0.001*			
Effect size (w)	0.567		0.838		0.799			

Different (uppercase letters) superscripts in the same column indicate statistically significant change by time

*TA* topical Triamcinolone acetonide, *Zn* systemic zinc, *VIT D* systemic vitamin D

^*^Significant at* P* ≤ 0.05

## Discussion

Zinc deficiency is estimated to affect billions of people worldwide, especially in the developing countries [31]. Zinc deficiency is linked to oral mucosal lesions such as recurrent aphthous stomatitis, oral lichen planus, burning mouth syndrome and atrophic glossitis [32]. Zinc plays a critical role in the pathogenesis of OLP by influencing epithelial growth, development, wound healing, and regeneration. Zinc deficiency disrupts the balance of T-helper lymphocytes, reducing TH1 cells and increasing TH2 cells, which can lead to cytotoxic effects on keratinocytes. Additionally, zinc regulates the production of pro-inflammatory cytokines, and its deficiency is associated with an excess of these cytokines [5, 17, 33–35]. Zinc has anti-inflammatory properties by targeting oxidative stress, which is implicated in OLP pathogenesis by inducing cellular damage and inflammation [20, 36]. Acting as a cofactor for antioxidant enzymes like superoxide dismutase, zinc facilitates the neutralization of reactive oxygen species (ROS), thus curbing oxidative damage. It also safeguards cell membranes against lipid peroxidation, preventing membrane injury, and aids in DNA repair, maintaining genomic stability. Zinc deficiency may disrupt redox balance, leading to uncontrolled ROS release during inflammation, potentially contributing to OLP [31, 33]. Several studies investigated the relationship between serum zinc levels and OLP, revealing a consistent pattern of reduced zinc levels in affected patients [37–39]. Studies assessing the therapeutic effect of supplementary zinc are scarce with only case report [39] and two clinical trials, assessing topical zinc as oral rinse [40] and systemic zinc as 50 mg tablet [30]. There are various biological forms of zinc for therapeutic and supplemental use. Zinc sulfate is the most commonly available form [30]. Octozinc, the zinc supplement used in our study, is a zinc sulphate supplement available in 25 mg oral tablets at a very affordable cost. In our study, the dose of zinc supplementation was 50 mg per day in compliance with the upper intake limit of zinc as advised by the US National Institutes of Health Office of Dietary Supplements [41]. In our study, among the three studied groups, supplementary zinc was the leading group in OLP management regarding pain and clinical improvement. While baseline pain scores were similar across the three groups, there were significant differences by Week 3, indicating that systemic zinc may offer a greater reduction in pain compared to topical triamcinolone acetonide. Regarding Thongprasom scores, supplementary Zn group showed statistically significant reductions between the baseline and the first week, followed by reductions between the second and third weeks, and finally between the seventh and eighth weeks, which consistently occurred one week earlier than in the control group, indicating that supplementary zinc may offer an earlier clinical improvement compared to topical steroids in OLP. Moreover, Group II (Zn) showed the most significant reduction among the studied groups, with a median Thongprasom score of 0 by Week 8.

The results of our trial are consistent with the previous trial investigating supplementary zinc in OLP management, conducted by Mehdipour et al., where they compared the efficacy of 0.2% zinc mouthwash with fluocinolone and fluocinolone alone in the treatment of OLP. They found that 0.2% zinc mouthwash with fluocinolone was effective to decrease lesion surface area, pain and burning sensation, attributed to the effect of zinc in healing the disrupted epithelium. Their results showed statistical significance in the intragroup comparisons only, with no significant difference in the intergroup comparison. Similar to our study, Mehdipour et al., continued the corticosteroid therapy all along the treatment period of the study, which was also an 8-week duration [40].

Suvarna et al. compared the efficacy of 0.1% triamcinolone Orabase and 50 mg oral zinc with triamcinolone. Both groups showed significant reductions in pain and lesion size in the intragroup comparison, which is similar to the results of our trial. However, in contrast to our study, Suvarna et al. discontinued the use of the steroid after one week to avoid potential side effects. In our study, oral steroid application was continued throughout the treatment period, and to address the drawbacks of long-term steroid use, topical antifungal treatment was prescribed [30].

Vitamin D deficiency is a worldwide health concern, its prevalence exceeds 80% in the middle east [42]. In Egypt, it is estimated to reach 100% among females and 88% among males [43]. Diverse roles of vitamin D are unveiling after long years of being restricted in its classical effect of maintaining calcium-phosphate balance and regulating bone metabolism, the effect of vitamin D is extending to include anti-carcinogenic effect, neuromodulating effect, cardioprotective effect, anti-infectious effect, anti-inflammatory and immunomodulating effects [44–46]. Vitamin D deficiency is linked to diverse oral immune-mediated disorders such as recurrent aphthous stomatitis, Behcet’s disease, lupus erythematosus, pemphigus vulgaris, and oral lichen planus [44–46]. The role of vitamin D in OLP pathogenesis is being revealed with time, vitamin D modulates the effect of monocytes, macrophages, and dendritic cells. It reduces pro-inflammatory cytokines including TNF-α, IFN-γ, IL-1β, IL-6, IL-8, and IL-17 and enhances anti-inflammatory cytokines such as IL-10. Furthermore, vitamin D has regulatory effect on keratinocyte proliferation and differentiation. Studies associated low level of vitamin D with increased mucosal inflammation and disruption of mucosal barrier [45–47].

Recent observational findings have highlighted potential systemic links between vitamin D deficiency and chronic inflammatory conditions. In a study by Chaitanya et al., melasma—a common hyperpigmentation disorder—was investigated within the context of a broader clinical presentation that maybe referred to as *Nallan C-Melasma Syndrome*. The study suggests a possible syndromic relationship involving shared pathogenic mechanisms between mucocutaneous disorders such as melasma, vitamin D abnormalities, and clinical periodontitis. These findings support the rationale for investigating adjunctive micronutrient therapies—particularly vitamin D and zinc—in managing chronic oral conditions like symptomatic oral lichen planus [48].

In light of the above, Vitamin D gained attention either in clinical trials [49–51] or observational studies [22, 52] as a treatment modality for OLP compared to standard steroid therapy. The populations studied varied between peri-menopausal women, post-menopausal women, or men, with doses ranging from 50,000 IU weekly, 60,000 IU weekly, 200,000 IU weekly, and duration ranging from 1 to 2 months.

The results of the current trial suggest that topical steroid with supplementary vitamin D may offer a greater reduction in pain and consistent clinical improvement compared to topical steroid alone in symptomatic OLP. These results are similar to the studies that have investigated the therapeutic role of supplementary vitamin D in OLP treatment. Razi et al. conducted one of the earliest studies on the efficacy of vitamin D supplementation in OLP treatment in peri-menopausal females [49]. They found significant pain reduction in the two studied groups (steroid therapy with Vitamin D supplementation versus steroid therapy) and significant clinical improvement in vitamin D group only between weeks 1 and 4, a pattern consistent with our study, where clinical signs and lesion size improved by weeks 4 and 8 compared to baseline. Our findings align with another study conducted by Shoukheba in 2020 [50], where intragroup comparisons revealed significant pain reduction in vitamin D group, however intergroup comparison did not demonstrate significant difference between vitamin D and topical steroids. For clinical improvement, results of the two groups were similar. Delavarian et al. documented that pain reduction was more prominent in vitamin D group and they detected a significant difference between the two studied groups (vitamin D supplements as an adjuvant to the conventional steroid therapy and or placebo) at the second and fourth weeks. Clinical improvement was statistically significant in vitamin D group only and a significant difference was detected between the two groups in the second week [51]. Furthermore, Gupta et al. and Nazeer et al. observed more significant improvement with vitamin D supplements versus standard steroid therapy in management OLP [22, 52].

Despite the growing interest in the benefits of vitamin D, there's no consensus on the appropriate dosage as supplementary use. Most of the recommended doses focus on optimizing bone health, but there's a lack of guidelines for other effects or health outcomes [53–56]. The aforementioned trials which examined the efficacy of supplementary vitamin D in managing OLP, used large doses of vitamin D due to their focus on patients with vitamin D deficiency and their ability to measure vitamin D serum levels [49–51]. In our trial, a lower dose of vitamin D (2,800 IU per week) was used, aligning with the recommended supplementary dose outlined in the National Institutes of Health Office of Dietary Supplements'Fact Sheet for Health Professionals in 2022 [57]. This decision was based on our inability to measure serum vitamin D levels due to budgetary and methodological constraints.

The assessment of plasma 25-hydroxyvitamin D [25(OH)D] and serum zinc levels before and after intervention to evaluate the biochemical impact of vitamin D and zinc supplementation was not performed in the present study due to significant financial and logistical constraints. Plasma vitamin D testing involves laboratory-based quantification of 25(OH)D using immunoassays or high-performance liquid chromatography-mass spectrometry, both of which are technically demanding and relatively costly [58, 59]. Similarly, accurate quantification of serum zinc typically requires sophisticated techniques such as atomic absorption spectrophotometry or inductively coupled plasma mass spectrometry [60, 61]. Given the limited funding available for our study, it was not feasible to routinely measure these biochemical markers. Moreover, measuring baseline levels of these elements prior to group allocation could have violated the randomization process by potentially introducing selection bias and stratification imbalances, thus compromising the integrity of the random assignment [62]. While we recognize that the absence of plasma 25(OH)D and serum zinc data represents a limitation, we prioritized clinically relevant outcome measures such as symptom severity (VAS score) and lesion improvement, which are validated and commonly used in oral lichen planus research [63].

To the best of our knowledge, the current study represents the first study to directly compare the supplementary effects of zinc (Zn) and vitamin D, compared to topical steroid therapy, in the management of OLP, and the second trial to assess the supplementary effect of systemic zinc in management of OLP.

Limitations of this study include the absence of serum zinc and vitamin D level monitoring in patients to validate the drug's efficacy in treating OLP. Future studies with more financial support should certainly consider integrating biochemical endpoints, including baseline and post-treatment plasma vitamin D and serum zinc levels, to further support clinical findings. Long-term follow-up with larger sample sizes is recommended to explore any potential recurrence trend.

## Conclusions

The superior efficacy of systemic zinc supplementation combined with topical corticosteroid application, in terms of pain reduction and clinical improvement, suggests its potential as an adjunct therapy for OLP management. Similarly, topical corticosteroid supplemented with vitamin D has comparable effect to topical corticosteroid alone on pain and clinical improvement in symptomatic OLP.

## Data Availability

The datasets used and/or analyzed during the current study are available from the corresponding author on reasonable request.

## Abbreviations

OLP: Oral lichen planus
WHO: World health organization
p53: Tumor protein p53
PCNA: Proliferating Cell Nuclear Antigen
REU system: Reticular-Erosive-Ulcerative REU system
ODSS: Oral Disease Severity Score
RCT: Randomized controlled clinical trial
VAS: Visual analogue scale
SD: Standard deviation
TA: Topical Triamcinolone acetonide
Zn: Systemic zinc
VIT D: Systemic vitamin D
Th1, Th17: T helper cell subtype 1 and 17
IL-2, IL-1β, IL-6, IL-8, and IL-17: Interleukins subtypes
TNF-α: Tumor necrosis factor α
IFN-γ: Interferon γ
MMP-9: Matrix Metalloproteinases
VDR: Vitamin D receptor
LPS: Lipopolysaccharide
[25(OH)D]: Plasma 25-hydroxyvitamin D
